# Factors Affecting the FcRn-Mediated Transplacental Transfer of Antibodies and Implications for Vaccination in Pregnancy

**DOI:** 10.3389/fimmu.2017.01294

**Published:** 2017-10-13

**Authors:** Christopher R. Wilcox, Beth Holder, Christine E. Jones

**Affiliations:** ^1^National Institute of Health Research Wellcome Trust Clinical Research Facility, Southampton, United Kingdom; ^2^Paediatrics Section, Division of Infectious Diseases, Centre for International Child Health, Imperial College London, London, United Kingdom; ^3^Faculty of Medicine, Institute for Life Sciences, University of Southampton, University Hospital Southampton NHS Foundation Trust, Southampton, United Kingdom

**Keywords:** neonatal Fc receptor, placenta, antibody, immunoglobulin G, pregnancy, maternal, vaccination

## Abstract

At birth, neonates are particularly vulnerable to infection and transplacental transfer of immunoglobulin G (IgG) from mother to fetus provides crucial protection in the first weeks of life. Transcytosis of IgG occurs *via* binding with the neonatal Fc receptor (FcRn) in the placental synctiotrophoblast. As maternal vaccination becomes an increasingly important strategy for the protection of young infants, improving our understanding of transplacental transfer and the factors that may affect this will become increasingly important, especially in low-income countries where the burden of morbidity and mortality is highest. This review highlights factors of relevance to maternal vaccination that may modulate placental transfer—IgG subclass, glycosylation of antibody, total maternal IgG concentration, maternal disease, infant gestational age, and birthweight—and outlines the conflicting evidence and questions that remain regarding the complexities of these relationships. Furthermore, the intricacies of the Ab–FcRn interaction remain poorly understood and models that may help address future research questions are described.

## Introduction

Despite medical advances, infection continues to be a leading cause of neonatal and infant morbidity and mortality worldwide (1). At birth, neonates encounter a wide range of new pathogens and have an inexperienced immune system, making them particularly vulnerable to infection (2). The transfer of antibodies from the mother to the fetus across the human placenta is central for providing immunity in early life. Vaccination in pregnancy is a strategy that aims to protect mother and infant by increasing the concentration of maternal vaccine-specific antibody, and thereby the quantity transferred to the infant by transplacental transfer (3). This serves to protect the newborn until the time of infant vaccination, or until the window period of greatest susceptibility has passed.

In the human placenta, a histological barrier separates the blood in the maternal and fetal circulations. This barrier consists of two layers: the multinucleated synctiotrophoblast and the endothelial cells of the fetal capillaries. Wide ranges of substances are transferred, either actively or passively, across the placenta from mother to fetus, including the nutrients and solutes needed for normal fetal growth and development. Many compounds of low molecular weight (<500 Da) will simply diffuse across the placental tissue, whereas substances of very high molecular weight are usually not able to transverse the placental barrier (4). One of the exceptions is immunoglobulin G (IgG), which has a molecular mass of 160 kDa, yet is actively transported from mother to fetus (5). Of the five antibody classes in humans, IgG is the only one to be transferred across the placenta in significant quantities, and this process begins at around 13 weeks of gestation (6).

Transplacental antibody transfer occurs *via* binding with the neonatal Fc Receptor (FcRn) in the placental synctiotrophoblast (7). A better understanding of mechanisms underlying FcRn-mediated transplacental antibody transfer, and the factors that affect these, is thus crucial for the optimization of maternal vaccination strategies, especially for developing countries where the burden of maternal and neonatal morbidity and mortality is highest (3). This review therefore sets out to summarize our current understanding of this field, review factors affecting FcRn-mediated transport of relevance to vaccination in pregnancy, and highlight gaps in our knowledge to direct future research.

## The Role of Vaccination in Pregnancy

Increasingly, vaccination in pregnancy is being recognized as a vital strategy to protect mother, fetus, and infant from infection and the associated adverse consequences. A number of vaccines are now routinely offered to pregnant women in several countries, including tetanus, influenza, and pertussis (8). Other vaccines may be offered to women in special circumstances (such as foreign travel and during outbreaks) and include meningococcus, inactivated poliovirus, and hepatitis A and B. Live vaccines are contraindicated in pregnancy. Vaccines currently progressing through the vaccine pipeline with a specific indication of use in pregnancy or pre-pregnancy include respiratory syncytial virus (RSV) (9), group B streptococcus (GBS) (10), and cytomegalovirus (11). Vaccination in the neonatal period is challenging as neonates may mount ineffective protective immunity, and the presence of maternal antibodies can blunt vaccine responses (2, 12).

Maternal vaccination is a highly effective approach to protect infants from infection. Early evidence comes from a study of tetanus vaccination in pregnancy in Papua New Guinea in the 1960s. Ten percent of infants born to mothers who received either no doses or one dose of tetanus developed neonatal tetanus compared to 0.57% of infants whose mothers had received three doses (13). More recent observational (14) and randomized controlled trials (RCTs) (15–17) conducted in both developed and developing countries have demonstrated that infants of influenza vaccinated mothers were 45–63% less likely to have episodes of proven influenza illness in early infancy (4–6 months of age). Furthermore, two of these RCTs showed that influenza vaccination reduced the incidence of maternal respiratory illness by 36 and 50.4% (15, 16). Maternal vaccination with a pertussis-containing vaccine is now routinely recommended in several countries and has been shown to be safe and to result in high concentrations of antibody in the infant over the first 2 months of life (18–21). Furthermore, maternal vaccination against pertussis has been demonstrated to have an effectiveness of over 90% at preventing disease in infants up to 3 months of age (22–24). Little is known regarding the beneficial effects of vaccination in pregnancy on breast-feeding, in which the transfer of secretory immunoglobulin A (IgA) antibodies serve to protect infants in the first few months of life by binding and opsonizing pathogenic microorganisms (25). However, recent studies have demonstrated that higher concentrations of secretory IgA to various diseases exist following maternal vaccination (26), with the strongest evidence coming from studies of influenza vaccination (27, 28).

Underpinning maternal vaccination is the effective FcRn-mediated transplacental transfer of vaccine-induced maternal IgG. A better understanding of the mechanisms of transplacental antibody transfer and the factors that affect this is crucial to optimize maternal vaccination strategies. Factors discussed below include IgG subclass, IgG glycosylation, maternal IgG concentration, maternal disease, gestational age at birth, and birthweight, all of which may all affect the protection conferred to the infant by maternal vaccination.

## IgG and the FcRn

**Figure 1:**
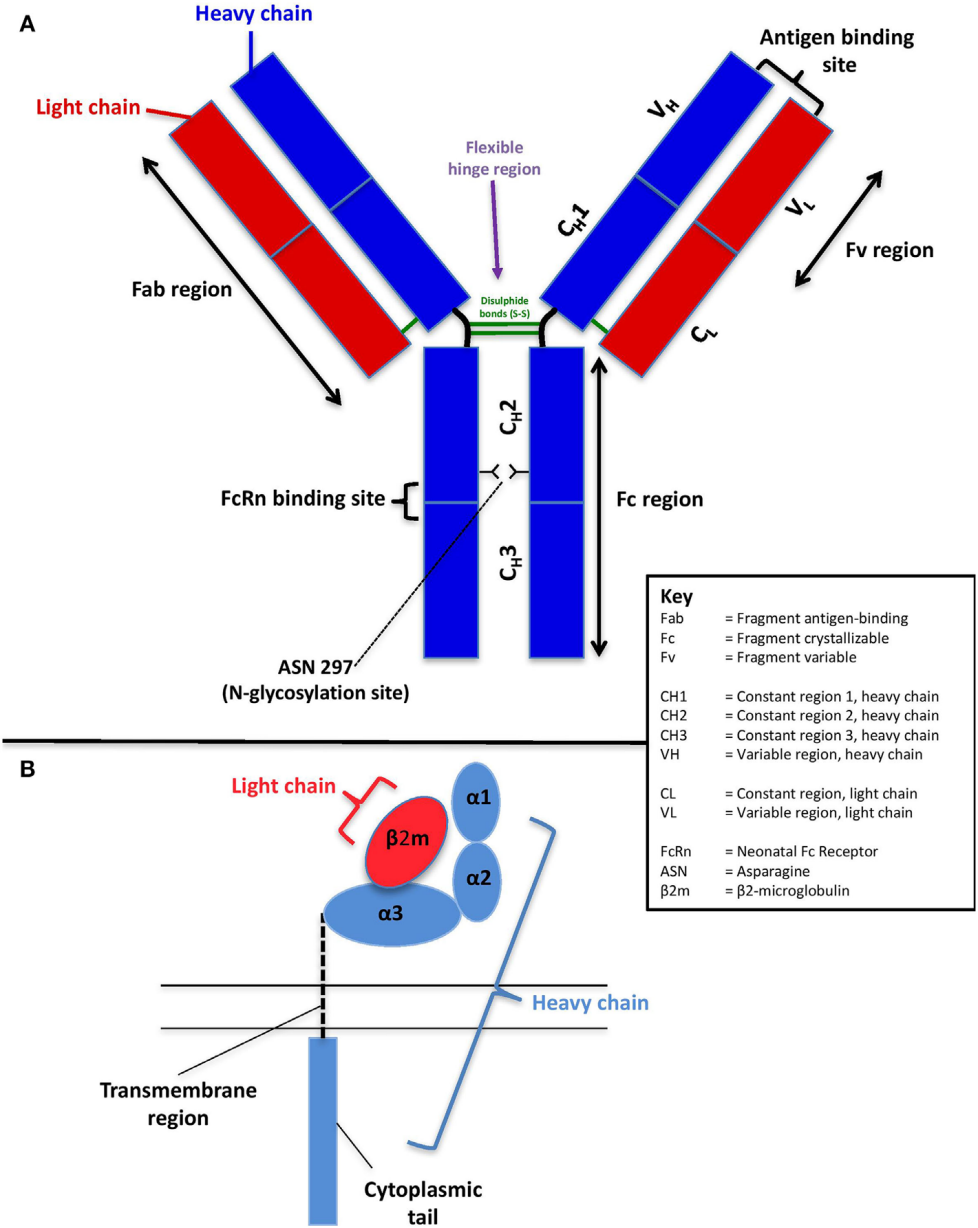
Schematic of the structure of human immunoglobulin G **(A)** and the neonatal Fc receptor **(B)**.

The human IgG molecule is a heterodimer of two identical 50 kDa heavy chains and two identical 23 kDa light chains (5) (Figure 1A). The heavy chains are of five different classes: μ, γ, δ, α, and ε, with four subclasses of γ and two of α. The light chains are of two classes: κ and λ (29). Together, the light and heavy chains form a Y-shaped structure, consisting of two fragment antigen-binding (Fab) arms, which contain the antigen-binding site and one crystallizable (Fc) tail region (30). The Fab region consists of constant and variable regions of the light chain, constant region 1 of the heavy chain (C_H_1), and variable region of the heavy chain (V_H_). Constant regions two and three of the heavy chain (C_H_2 and C_H_3) form the fragment crystallizable (Fc) tail region (30). A flexible hinge of disulfide bonds connects the C_H_1 and C_H_2 domains, to allow the Fab arms freedom of movement from the fragment crystallizable (Fc) tail. The outward-facing part of the interface between the C_H_2 and C_H_3 domains is where binding with FcRn occurs.

On the basis that whole IgG molecules and the Fc portion of IgG pass into the fetal circulation more readily than antigen-binding Fab fragments, it was hypothesized in the 1960s that receptors for the Fc part of IgG (FcγR) may be involved in the placental transfer of IgG (31). A functionally distinct FcγR was first proposed to mediate this specific transport of IgG by Brambell (32, 33), and this was later established to be the neonatal Fc receptor (FcRn)—termed as such due to its identification in the gut epithelial cells of neonatal rats (34). Its existence was confirmed by further work in mice (35, 36), and direct evidence of its involvement in the delivery of maternal IgG came from *ex vivo* perfused placenta studies comparing the maternofetal transfer of a recombinant IgG1 with that of a variant containing a mutation in the Fc region that did not bind to FcRn (37).

The structure of FcRn is unlike other Fc receptors and is markedly similar in structure to major histocompatibility complex (MHC) class I, with which it shares 22–29% sequence homology (37) (Figure 1B). It is a heterodimer consisting of a complex of two chains: a polypeptide α-chain (heavy chain) and β2-microglobulin (light chain) (38). The heavy (45 kDa) α-chain is encoded on chromosome 19 and consists of three extracellular domains (α1, α2, and α3), a transmembrane region, and a short cytoplasmic tail. While the α-domains are closely related to MHC class I, the transmembrane and cytoplasmic domains distinguish FcRn from other receptors of the same class (39). The light (12 kDa) chain, β2-microglobulin (β2m), is encoded on chromosome 15 and is non-covalently associated with the α3 domain (38).

The role of FcRn extends beyond its role in placental FcRn transport. It is central to the homeostatic maintenance of both serum IgG and albumin levels by protecting them from lysosomal degradation and is thereby responsible for their long serum half-lives relative to other plasma proteins (40, 41). Furthermore, FcRn is increasingly recognized to have a wide role in modulating humoral and cell-mediated immunity (42). It is involved in the bidirectional transcytosis of IgG and IgG immune complexes across various human epithelia (43–45), and its expression in hematopoietic cells (46, 47) is essential for the enhancement of IgG-mediated phagocytosis (48), anti-tumor immunosurveillance (49), and the direction of immune complexes to lysosomes in dendritic cells in order to facilitate antigen presentation (50, 51).

## Mechanisms of FcRn-Mediated IgG Transcytosis in the Placenta

**Figure 2:**
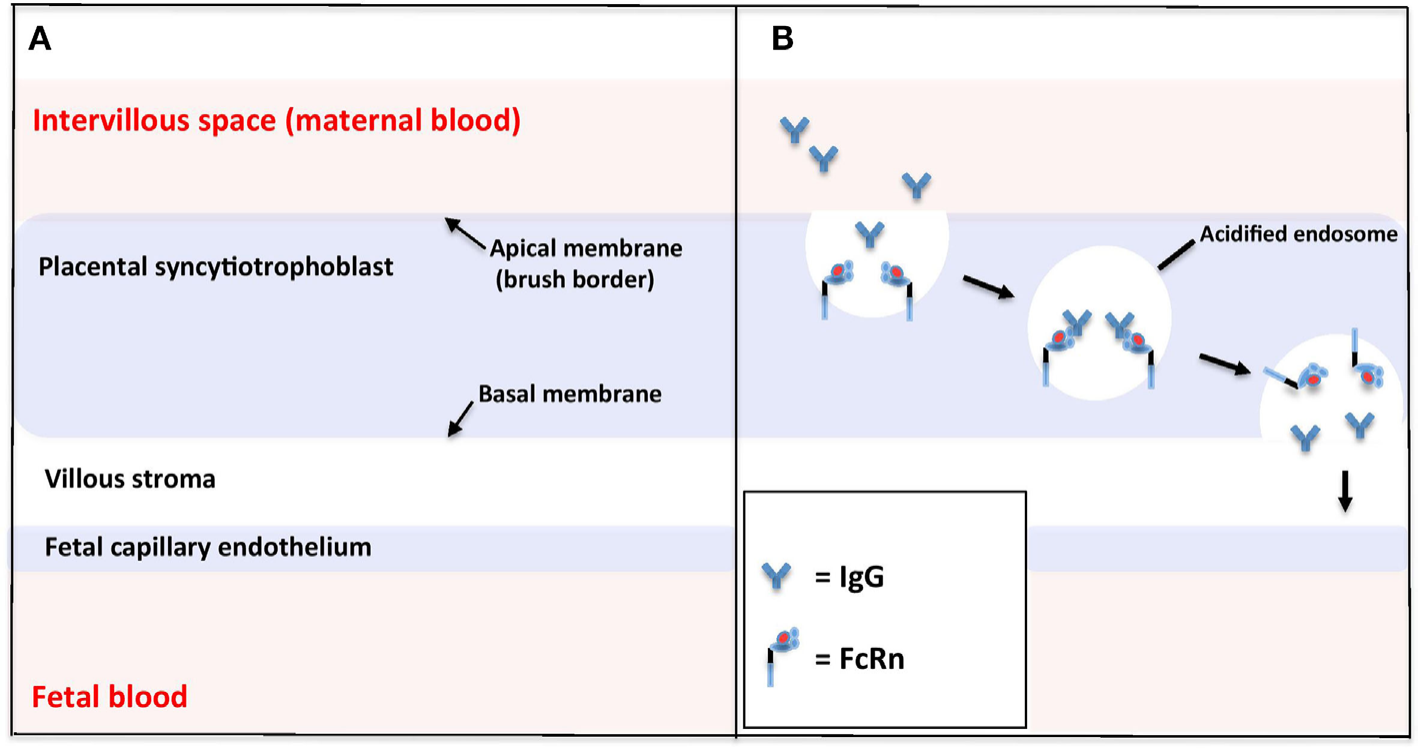
Schematic of the microstructure of the human placental barrier at term **(A)** and the neonatal Fc receptor (FcRn)-mediated endocytosis of immunoglobulin G (IgG) across the placental syncytiotrophoblast **(B)**.

The placenta is a complex organ of which the basic functional unit is the chorionic villus (52, 53). Villi are highly branched vascular projections of fetal tissue, through which fetal blood flows from the umbilical cord. The villi are surrounded chorion, which consists of two layers: the outer syncytiotrophoblast (which is in direct contact with maternal blood flowing through the intervillous space) and the inner layer of cytotrophoblast progenitor cells. Under the chorion lies the stroma and the fetal capillaries (Figure 2A).

Unlike other FcγRs, the interaction of FcRn with IgG displays a strong pH dependence, such that high-affinity binding occurs at pH 6.0, but little or no binding occurs at physiological pH 7.4 (54). This pH-selective binding is key to the effective transport of IgG across the synctiotrophoblast of the placenta from the maternal to fetal circulation. Various crystallography studies have found structural modifications in the FcRn α-chain that might contribute to this pH dependence. This characteristic is likely mediated, at least in part, *via* protonation of histidine residues (the only amino acid that changes between pH 5.5 and 7.4) at the C_H_2–C_H_3 domain interface of IgG (54–56). Additionally, thermal denaturation studies have shown that the FcRn heterodimer is significantly more stable at pH 6 than pH 8 (57).

To be successfully transferred across the placenta, maternal IgG must cross the synctiotrophoblast layer, the villous stroma, and the fetal vessel endothelium. The mechanisms of FcRn-mediated IgG trancytosis across the synctiotrophoblast have been elucidated by the use of the BeWo choriocarcinoma cell line (a model for placental trophoblast) (58) and fluorescence microscopy of FcRn-green fluorescent protein-transfected live human endothethial cells, which enable analysis of the intracellular trafficking of IgG in real time (Figure 2B) (59, 60). These studies suggest that IgG is taken up from the extracellular fluid on the apical side of the synctiotrophoblast by endocytosis. Within the acidic environment of endosomes, IgG binds with membrane-bound FcRn and is protected from proteolytic degradation by lysosomal enzymes. IgG is then transcytosed to the basal cell surface, where a return to physiological pH causes dissociation of IgG from FcRn. FcRn may then be recycled back to the maternal membrane to perform more cycles of transcytosis.

The mechanisms underlying the initial endocytosis of IgG, and onward transport of IgG across the villous stroma and the fetal vessel endothelium remain somewhat of a gap in our knowledge. It is controversial as to whether FcRn is also expressed in fetal vessel endothelium. Various studies using immunohistochemical staining of placental sections with anti-FcRn antibodies have shown a mix of some (61, 62) or no (36). FcRn expression in fetal endothelium, and some evidence, points toward alternative Fc receptors in the further movement of IgG (63, 64).

## Factors Associated with Changes in Transplacental Antibody Transfer

### How Does the Structure of IgG Vary between Subclasses and How Might This Affect FcRn Binding and Transplacental Transfer?

Human IgG can be divided into four subclasses (IgG1, IgG2, IgG3, and IgG4), named in order of decreasing abundance (65). IgG subclasses are over 90% identical at the amino acid level; however, each subclass has a unique functional profile. In human serum, FcRn prolongs the half-life of IgG1, IgG2, and IgG4 equally. It is thought that FcRn does not prolong the half-life of IgG3 in the same way, because IgG3 has an arginine at position 435 instead of the histidine found at the same position in the other subclasses, except for individuals expressing a natural IgG3 variant (H435) (66). IgG1 is preferentially transported across the placenta, followed by IgG4, IgG3, and IgG2 (37, 67). Placental IgG transport has been estimated by comparing cord and maternal concentrations of IgG subclasses. These studies have shown that concentrations of IgG1, IgG4, and H435-containing allotypes of IgG3 exceed maternal levels; however, levels of IgG2 do not (68–70). This suggests that the placental transport of IgG2 is significantly less efficient.

One explanation for this difference in placental transport relates to the IgG hinge region, as differences in the length and flexibility of the hinge region are found in the subclasses. The length and flexibility of the hinge region affects the orientation and movement of the Fab arms in relation to the Fc tail (5). The relative flexibility differs as follows: lgG3 > lgG1 > lgG4 > lgG2 (71). IgG2 has been demonstrated to have a uniquely short hinge region, comprising 12 amino acids and containing a poly-proline double helix, stabilized by four inter-heavy chain disulfide bridges (72). This causes the Fab arms to be relatively close to the Fc tail and enables its κ isotope, but not λ isotope, to form three disulfide isoforms that differ from each other with relation to their disulfide bridges in the hinge region (73). It has therefore been postulated that one these IgG2κ isoforms may have decreased interaction with FcRn and account for the reduced placental transport displayed by IgG2. However, recent studies in humans have found that FcRn binding does not seem to vary among these different disulfide isoforms (74) and that no preference occurs for recycling and placental transport of IgG2λ or IgG2k (69).

The question therefore remains over the mechanism underlying the reduced placental transport of IgG2 relative to other subclasses. One possible explanation relates to recent findings suggesting that different proteins are involved in regulating FcRn-mediated IgG transcytosis (actin motor myosin Vb and Rab25) and recycling (Rab11a), respectively (75). While IgG2 transport through the placenta is indeed low, its recycling and half-life extension in the adult circulation are even better than IgG1 (69). It is therefore a possibility that regulation by these proteins varies depending on IgG subclass, but how the stoichiometry of IgG2 may affect these intracellular processes requires further study. Another possible explanation is that another Fc receptor, FcγRIIb, may play a role in modulating transplacental antibody transport (76, 77). A role for FcγRIIb might provide a plausible explanation for the reduced transport of IgG2 because, unlike other subclasses, IgG2 has almost non-existent binding affinity to FcγRIIb (78).

The discrepancy between transfers of different IgG subclasses may have key implications for maternal vaccination. IgG2 is crucial for the opsonization and killing of polysaccharide-encapsulated pathogens and is induced by polysaccharide vaccines (69). Conversely, vaccines that contain protein antigens, such as tetanus, predominantly elicit production of IgG1 and IgG3. Therefore, transcytosis of some vaccine-induced IgG subclasses is more efficient than others. Future work to optimize placental transfer of IgG2 has the potential to better protect infants against important polysaccharide-encapsulated pathogens such as GBS, Haemophilus influenzae B (HiB), and *Neisseria meningitidis* (79).

### How Does Glycosylation of IgG Affect FcRn Binding and Transplacental Transfer?

Glycosylation involves the covalent addition of sugar moieties (such as fructose, galactose, and sialic acid) to proteins. The dynamics and binding affinity of IgG can be influenced by its glycosylation (80), and IgG exists in a number of glycosylated variants (glycoforms) (81). Both pregnancy and disease may have an impact on IgG glycosylation. Pregnancy is associated with increased Fc and Fab region galactosylation and sialylation (82). Interestingly, pregnancy is also associated with clinical improvement of autoimmune disease (such as rheumatoid arthritis), which, as well as infectious disease, is associated with a reduction in galactosylation of IgG in human serum (83).

Neonatal Fc receptor binds to the outward-facing part of the C_H_2 and C_H_3 domains of the Fc region of IgG. The *N*-glycosylation site occupies the inner part of the Fc region at asparagine 297, helping to maintain its quaternary structure and stability [Figure 1A; Ref. (84)]. It has therefore been suggested that IgG glycosylation may affect the IgG–FcRn interaction and that that there may be a preferential placental transport for glycosylated IgG. Supportive evidence for the hypothesis of preferential transport of glycosylated IgG comes from studies in the 1990s, which demonstrated reduced concentrations of non-glycosylated IgG and higher concentrations of galactosylated IgG in newborn infants (85, 86). More recently, Dashivets et al. studied enzymatically engineered glycosylation variants and showed that deglycosylated IgG1 had a slightly diminished binding to FcRn, with digalactosylated IgG demonstrating superior binding than monogalactosylated and agalactosylated variants (87). Furthermore, *in vivo* pharmacological studies have also shown an impact of the glycan on the half-life mediated by FcRn (88).

Evidence to the contrary, however, includes a study by Bakchoul et al. that showed agalactosylated IgG was transported equally well across the placenta (89). In addition, Einarsdottir et al. studied Fc region glycosylation for all IgG subclasses in 10 pairs of fetal and maternal IgG samples. They demonstrated comparable Fc region glycosylation for all IgG subclasses (including galactosylation, sialylation, bisecting G1cNAc, and fucosylation), suggesting that transplacental IgG transfer does not favor certain Fc glycoforms (90). However, another more recent study by the same group in 2016 found clear, albeit minor, differences in the *N*-glycosylation profile of IgG between maternal and umbilical cord plasma in 42 mother–newborn pairs (91). Levels of galactosylation were slightly higher for cord IgG, with lower levels of bisection, sialylation, and sialylation per galactose. Possible reasons for the differences observed between studies include a IgG subclass-related transport bias (discussed previously), as well as the method of measurement, which was at the released glycans level in the 2016 study, rather than by analyzing IgG-derived Fc-glycopeptides (92). It is therefore possible that it is the quality of Ab glycosylation, rather than the total quantity of glycosylation that determines transplacental transfer. It is not known how vaccination in pregnancy might affect glycosylation of IgG and the efficacy of transplacental transfer of vaccine-specific IgG and is an area where more research is needed.

### How Does Total Maternal IgG Concentration Affect Transplacental Transfer of Specific IgG?

It is well established that maternal antibody levels play a role in determining transfer efficiency. Neonatal IgG levels usually correlate with maternal ones; however, it has been suggested that once maternal total IgG levels reach a threshold (>15 g/L), FcRn can become saturated (37, 93). IgG must then compete for a finite number of FcRn receptors. Unbound IgG molecules are subsequently destroyed through the lysosomal degradation process within cells. This is supported by African studies showing that reduced IgG transfer ratios were associated with the higher maternal total IgG levels (94, 95). Furthermore, a number of more recent studies have demonstrated negative correlations between maternal IgG levels and placental transfer ratios for both total and antigen-specific IgG (96–98).

Very high concentrations of vaccine-specific antibodies could potentially result in a reduced proportion of maternal IgG being transferred across the placenta to the infant, resulting in a lower transplacental transfer ratio. However, the concentration of antibody in cord blood is still likely to be significantly higher in infants born to vaccinated women compared to infants born to unvaccinated women and therefore may not have implications for protective infant immunity, and to date, no adverse clinical outcomes have been observed.

### How Does Maternal Disease Affect the Ab–FcRn Interaction and Placental Transfer of IgG?

#### Maternal Infectious Disease

It is now well established that maternal chronic infection can reduce the transplacental transfer of IgG specific to a variety of important childhood pathogens, including RSV, measles, tetanus, and HiB (37, 99–103). The majority of these studies have focused on placental malaria and HIV, which are particularly prevalent in developing countries and continue to exert a significant burden of morbidity and mortality globally. These include studies of HIV-exposed but uninfected infants, which showed reduced transplacental transfer ratios and lower concentrations of specific antibodies than HIV-unexposed infants did to HiB, pertussis, pneumococcus, and tetanus at birth (104).

The mechanisms behind this reduced transfer are poorly understood, and current models remain speculative. Infections may impact on IgG transfer directly through infection and inflammation of the placenta, or a reduction FcRn-antibody binding avidity, or as detailed above, *via* induction of hypergammaglobulinemia (IgG > 15 g/L) leading to saturation of placental FcRn (105). Studies assessing the impact of infection and hypergammaglobulinemia have had a great deal of overlap between these populations (>90%), complicating the interpretation of these effects independently (95). One Malawian study demonstrated that reduced antibody transfer in placental malaria may occur independently of hypergammaglobulinemia using multivariate regression analysis (106); however, more recent conflicting evidence from Papua New Guinea showed that only hypergammaglobulinemia, and not placental malaria, was associated with impaired transport of RSV antibody (99). Further studies are therefore clearly needed to understand the complexities of these relationships.

Interestingly, non-pregnant individuals with infectious diseases such as HIV have been shown to have significantly higher levels of galactose-deficient IgG than healthy controls. If glycosylation does indeed impact on the Ab–FcRn interaction as discussed above, then this may represent a further possible mechanism by which HIV could impact on placental IgG transfer and thus the effectiveness of maternal vaccination (107, 108).

#### Maternal Nutrition and Non-Communicable Diseases

Maternal malnutrition can have adverse implications for the neonate, and it has been demonstrated that neonatal immune responses may be modulated by the nutrition of a mother during gestation (108). One study reported a 14% reduction in antibody transfer among malnourished pregnant women compared to controls (109); however, the reasons for this are unclear and possibly relate to differences in placental size, morphology, and vascular development (110, 111). Other studies of micronutrients include a recent review of antenatal zinc supplementation that did not find significant evidence for the positive effect of zinc on antibody transport (112).

Another significant maternal morbidity is diabetes mellitus, which can either be pre-existing or gestational and affects 0.2–0.3 and 2–5% of pregnancies, respectively (113). To date, the effect of maternal hyperglycemia on FcRn and IgG transfer remains unclear. Stach et al. (98) demonstrated an increased rate of IgG transfer in hyperglycemic mothers for all antigens they studied (GBS, Klebsiella LPS, and Pseudomonas LPS), as did França et al. (114). More recently, De Souza et al. investigated both the transfer of IgG and expression of FcRn expression (measured by flow cytometry), in normo- and hyperglycemic mothers (115). They found that mothers with pre-existing type 2 diabetes had lower total levels of IgG, and reduced leukocyte FcRn expression across maternal blood, cord blood, and placental samples (collected at delivery) compared with normoglycemic mothers. Interestingly however, FcRn expression increased with mild gestational hyperglycemia. There was no statistically significant difference in total IgG levels in newborns between groups of mothers. Differences were observed on subclass analysis however, with significantly lower transfer of IgG1, IgG3, and IgG4 in women affected by diabetes, but significantly higher transfer of IgG3 in women with mild gestational hyperglycemia.

This decrease in FcRn expression may explain the reduced transfer of some IgG subclasses in mothers with diabetes. Furthermore, high levels of glycated IgG have been demonstrated in the plasma of patients with diabetes, and this may have an effect on the avidity of binding with FcRn and its transfer across the placenta (116, 117). The question also remains over why higher transfer might occur for IgG3 in the context of mild gestational hyperglycemia. Hyperglycemia is associated with a variety of alterations to placental structure, including increased numbers of glucose transporters (118) and a discontinuity in the trophoblastic layer (119), which may both facilitate the passage of glucose, and possibly some immunoglobulins, across the placenta (120). Additionally, greater placental villous capillarization has been noted in women with mild gestational hyperglycemia, and may facilitate placental transfer of a variety of substances (121).

Another common complication in pregnancy is maternal hypertension, affecting 2–3% of pregnancies (122). One study has examined the effect of pregnancy-induced hypertension on IgG transfer and, interestingly, found that hypertension was associated was increased transfer of IgG against *Klebsiella* spp. (98). This might be considered paradoxical given the immune-pathological damage observed in the placenta of hypertensive women (123).

Clinical trials of vaccination in pregnancy typically enroll healthy women, without chronic infections or co-morbidities. As these factors may influence transplacental transfer of antibody and therefore the protection afforded to the infant, it is important to also design studies, which assess vaccines in pregnancies in “real-life” settings, without the extensive exclusion criteria applied to early phase clinical trials. These data also suggest that optimization of maternal health for the benefit of mother and infant is important.

### How Does the Ab–FcRn Interaction Change across Gestation and Birthweight?

Placental transfer of IgG occurs in an exponential fashion as pregnancy progresses, with minimal transfer in the first trimester (6). In the second trimester, the use of cordocentesis has demonstrated that fetal IgG rises from roughly 10% of the maternal concentration at 17–22 weeks of gestation, to 50% at 28–32 weeks (124). In the third trimester, the rate of IgG transfer rises significantly (particularly from 36 weeks), with the increase of fetal IgG concentrations between 29 and 41 weeks of gestation doubling that of 17–28 weeks. At term, fetal levels vary, however, usually exceed maternal levels by 20–30% (64, 125, 126).

It follows therefore that a reduced transfer of IgG in preterm infants compared with term infants has been demonstrated for a variety of pathogens (97, 127, 128) particularly for infants born at less than 36 weeks of gestation (126). This knowledge has significant implications for the optimal timing of vaccination in pregnancy and has shaped the development of maternal vaccination strategies, reviewed by Calvert et al. (129). In order to protect preterm infants, a vaccine would need to be given early in pregnancy to ensure sufficient time of transport of IgG to the infant. However, later vaccination could be more desirable to more closely match the peak antibody response with the peak of transplacental transport of IgG to the infant. There remains debate in the published literature about the optimal timing of vaccination in pregnancy. It is worth noting that, given the increased susceptibility of premature infants to serious early-life infections, the optimal strategy may require a compromise between giving the best protection to term babies, versus protecting all viable infants.

Birthweight may also affect IgG transfer, with studies demonstrating a reduced transfer of antibodies in term low birthweight infants (65, 130). Interestingly, on subclass analysis, the reduced transfer seen in premature and low birth weight infants has been shown to be specific to IgG1 and IgG2, which may in part explain the higher susceptibility of premature infants to infections caused by polysaccharide-encapsulated pathogens which predominantly elicit IgG2 production, such as GBS (97, 127).

It is thought that this change in rate of transplacental transfer may partly occur because of increased expression of FcRn throughout gestation; however, this is yet to be formally demonstrated and our understanding of the evolving expression of FcRn remains poor. Whether alternations in the Ab–FcRn interaction may also play a role in this effect is unknown. It is worth noting that preterm labor and low birthweight are associated with numerous maternal pathologies, such as gestational hypertension, diabetes, and preeclampsia, which may also have a direct or indirect effect on placental function and the Ab–FcRn interaction. Thus, interpreting their independent effects may therefore be challenging.

## What Models of Placental Function are Currently Available to Study Transplacental Transfer of Immunity?

Over the years, several models of placental function have been developed to study the transplacental transfer of substances, including IgG. Mouse and rat models have been central to the discovery of FcRn (34) and have provided useful insights into the possible mechanisms of FcRn-mediated IgG transfer in situations where human studies are considered invasive or impractical (131). However, they differ from humans in many key features including levels of FcRn expression (132), immunological function (133), and placental anatomy (77). Another major model has been paired maternal–cord samples, which have been used widely and offer the possibility of comparing blood samples from the mother at the time of delivery with umbilical cord blood. The ratio of cord:maternal antibody concentration has been used as a proxy for placental transport (104).

In addition, several *ex vivo* and *in vitro* placental models are available to study transplacental transfer at a more mechanistic level. The cell line most commonly used is the choriocarcinoma-derived BeWo (b30) cell line, which can be cultured to form polarized, confluent monolayers with tight junctions for use in directional transport studies. These trophoblast cells serve as an *in vitro* model of the rate-limiting barrier of maternal–fetal exchange and can be used to study placental metabolism and transport of numerous substances, including IgG (134–136). BeWo cells also demonstrate hormone secretion properties and characteristics of third trimester trophoblasts; however, the model lacks connective tissue and fetal endothelium, which are present in the *in vivo* human placenta. Also, as single cytotrophoblast cells with tight junctions, they do not fully recapitulate the multinucleate syncytiotrophoblast, which is the cell type in contact with maternal blood. Forskolin treatment has sometimes been used, as it can induce fusion of BeWos to form syncytia (137). However, this fusion is variable and never reaches 100%, so is unable to create a complete syncytiotrophoblast barrier for transfer studies. Culture of isolated primary term cytotrophoblasts, which differentiate in culture to model syncytiotrophoblasts can be employed to overcome this issue (138).

The gold standard for placental transfer studies is the placental perfusion model. For this, a term placental cotyledon is cannulated and re-perfused to model the fetal and maternal circulations, enabling the study of the placental transfer of a chosen substance (139). Compared to placental transfer *in vivo*, this model is obviously simplified and does not take into account some of the possible maternal/fetal physiological variables. It does however offer the best technique to study the transplacental exchange of substances across the intact human placenta (134, 140).

The BeWo and placental perfusion models have shown good comparability in studies comparing the transport of different compounds across the placenta in terms of rank order; however, the transfer rate is much slower in BeWo cells (141, 142). This could be due to the higher pressure and flow in the circulation pump setup of the perfusion model, and while the BeWo cell monolayer can be placed on a shaking plate to create flow, this is not in the same magnitude as the placenta and there is a lack of hydrostatic pressure on the fetal side (140). Also, as mentioned above, the BeWo is a cytotrophoblast model, not a syncytiotrophoblast model, and thus, uptake rates and receptor expression may differ. Despite its limitations, the BeWo model is far less technically challenging to perform than the perfusion model, which requires very rapid access to fresh placenta samples and has a high failure rate (134). It therefore may present a useful first-step model for those wishing to investigate placental transfer, before progressing to the more complex placental perfusion model, particularly for the study of inter-individual differences or disease states (139, 143).

One other consideration is that both the BeWo model and the placental perfusion models only enable the modeling of term placenta. This represents a gap in our knowledge, particularly as maternal vaccines are often given in the first and second trimester. One way to overcome this issue could be through the use of the placental explant model. For this, small placental villous explants are dissected and cultured *in vitro*. This model can be performed with placental tissue of any gestation and thus is a commonly used model for early placental function, as samples are obtainable from termination of pregnancies. The explant model enabled the first demonstration of Zika virus infection of the first trimester placenta *in vitro* (144) and has been used to investigate placental uptake of other substances, including glucose (145), amino acids (146), and exosomes (147). The explant model has not been extensively used for antibody investigations, except for in the study of antiphospholipid antibodies (148); this is likely due to the fact that it does not fully model maternal to fetal transfer. Nevertheless, the ability to demonstrate uptake into intact human placental tissue from across gestation could provide useful information regarding maternal antibody uptake and interaction with the FcRn throughout pregnancy, both requisite steps for transfer of antibody to the fetus.

## Conclusion

Since its first identification in 1989, it has become increasingly apparent that FcRn plays a lifelong role in immunity. Importantly for neonates, FcRn is crucial for establishing humoral immunity *via* transplacental IgG transfer, and this exciting research field continues to expand.

**Figure 3:**
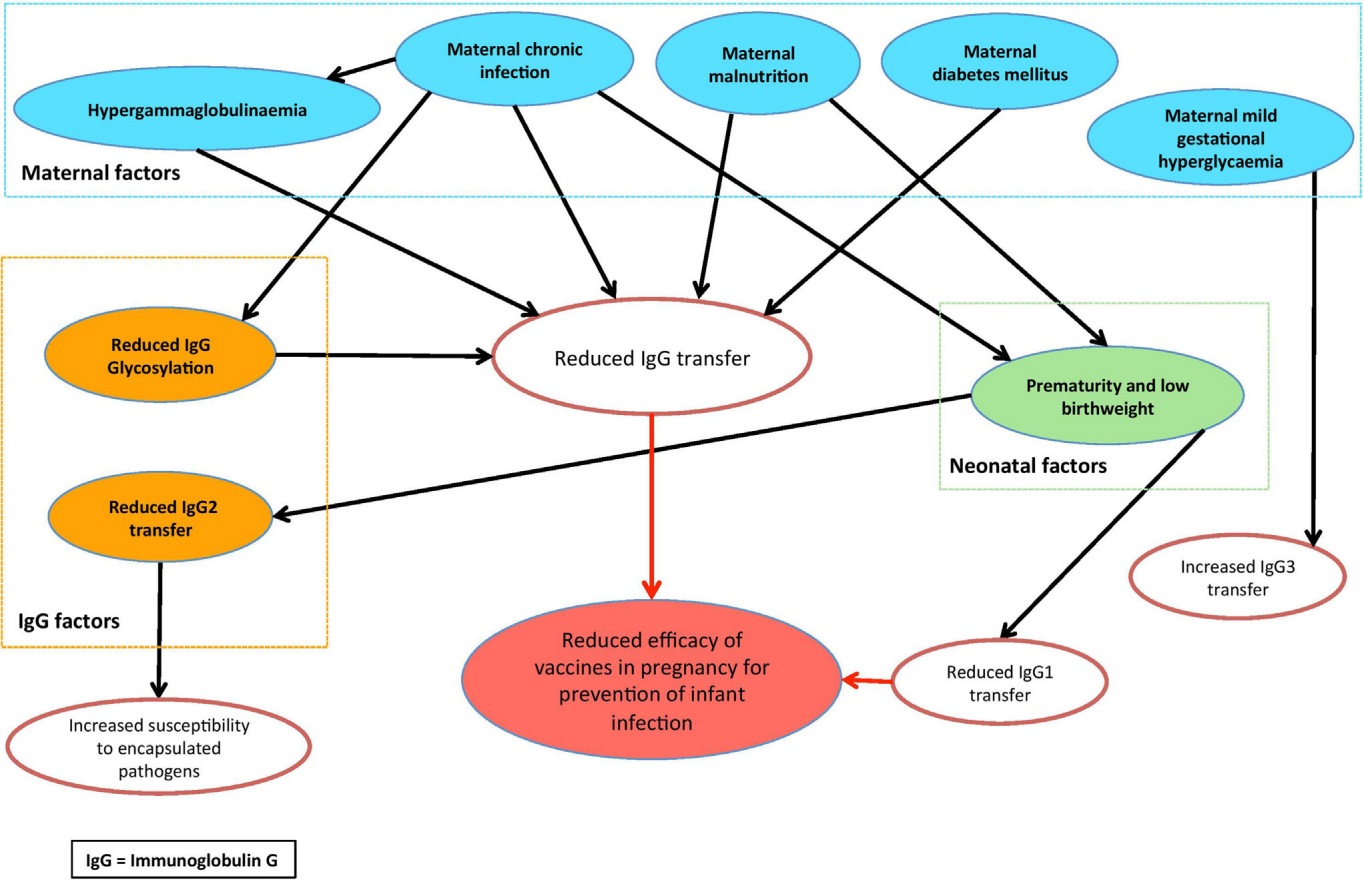
Conceptual diagram of the factors that may modulate placental antibody transfer of relevance to maternal vaccination.

This review has highlighted a number of factors that may affect the effective FcRn-mediated transplacental antibody transfer, which are summarized in Figure 3. These include IgG subclass, IgG glycosylation, maternal IgG concentration, maternal disease, gestational age at birth, and birthweight—yet there is conflicting evidence and many questions remain regarding the complexities of these relationships. Furthermore, while the role of FcRn in IgG transfer is well recognized, the intricacies of the Ab–FcRn interaction and how binding varies across subclass, gestation, glycosylation, and disease states remain poorly understood. Future research platforms will therefore benefit from utilizing a combination of placental models, as well as affinity studies of Ab–FcRn binding using approaches such as surface plasmon resonance and biolayer interferometry (149, 150), which represent an exciting new avenue for research.

As maternal vaccination becomes an increasingly important strategy for the protection of young infants, improving our understanding of the mechanism of transplacental antibody transfer and thus the factors that could impact vaccine effectiveness will be increasingly important, especially in developing countries where the burden of morbidity and mortality is highest.

## Author Contributions

CW designed and wrote the article. BH designed and critically revised the article. CJ (senior author) conceived, designed, and critically revised the article. All authors approved the final copy of the manuscript.

## Conflict of Interest Statement

The authors declare that the research was conducted in the absence of any commercial or financial relationships that could be construed as a potential conflict of interest.
